# Valid and accepted novel bacterial taxa isolated from domestic companion and agricultural animals described in 2024

**DOI:** 10.1128/jcm.01069-25

**Published:** 2025-10-17

**Authors:** Sara D. Lawhon, Claire R. Burbick, Trinity Krueger, Elena Ruiz-Reyes, Ayden Wisney-Leonard, Mallory Lenmark, Erik Munson

**Affiliations:** 1 Department of Veterinary Pathobiology, Texas A&M University 14736, College Station, Texas, USA; 2 Department of Veterinary Microbiology and Pathology, Washington State University 6760, Pullman, Washington, USA; 3 Department of Medical Laboratory Science, Marquette University 5505, Milwaukee, Wisconsin, USA; 4 Wisconsin Clinical Laboratory Network Laboratory Technical Advisory Group, Madison, Wisconsin, USA; Medical College of Wisconsin, Milwaukee, Wisconsin, USA

**Keywords:** taxonomy, nomenclature, veterinary microbiology

## Abstract

Recognizing and updating bacterial names is key to communication between the veterinary clinical microbiology laboratory, veterinarians, and clients. *Moraxella oculi* sp. nov., distinct from *Moraxella bovis* and *Moraxella bovoculi*, was isolated from a cow with infectious bovine keratoconjunctivitis. Several respiratory pathogens were recognized, including *Neisseria leonii* sp. nov. described from rabbits, *Mannheimia indoligenes* sp. nov. described from cattle in Europe, and *Moraxella haemolytica* sp. nov. described from a goat in China. *Stenotrophomonas forensis* sp. nov. is a novel designation within the *Stenotrophomonas maltophilia* complex associated with isolates derived from horses. New additions to the *Campylobacter* genus included *Campylobacter californiensis* sp. nov.*,* recovered from bovine feces during a raw milk-associated outbreak of campylobacteriosis in humans. Taxonomic revisions were most notable in Gram-positive organisms. A species within genus *Jeotgalicoccus* has been renamed, and the subspecies designation *Kocuria rosea* subsp. *polaris* comb. nov. has been created. *Streptococcus suis* was revised to *Streptococcus suis* subsp. *suis* subsp. nov. in recognition of the initial description of *Streptococcus suis* subsp. *hashimotonensis* subsp. nov., which was isolated from people in Japan with bite wounds from boars. *Chlamydophila caviae*, which causes respiratory disease and abortion in guinea pigs and is zoonotic, has been revised to *Chlamydia caviae* comb. nov.

## INTRODUCTION

Nomenclature and taxonomy remain central to scientific and clinical communication, creating consensus between disparate fields that draw upon the microbiological sciences and building a deeper understanding of the microbiological world that surrounds human and veterinary patients. Microbiome research has significantly impacted and expanded our knowledge of the bacterial species living in the unique ecological niches in host animals (1). This, combined with the potential impact on clinical communication that name changes such as those made to or directed at *Clostridioides difficile* (2–4) and *Rhodococcus equi* (5–8), further highlights the importance of taxonomy to clinical veterinary microbiologists.

In early 2023, the *Journal of Clinical Microbiology* launched a series of publications describing taxonomic changes and new species taxa relative to the field of veterinary microbiology (9–11). In an attempt to assist veterinary microbiology laboratories in staying current with taxonomic status of bacteria, the *Journal of Clinical Microbiology* has committed to publishing taxonomy updates on an annual basis, relative to bacterial species derived from domestic (12, 13) and non-domestic (14, 15) animal hosts. This frequency of publication is designed to contribute to expedient implementation of correct nomenclature into veterinary microbiology practice, as outlined in a recent practice guideline (16). In total, these reports have summarized accepted taxonomic announcements dating back to 2018. The current report summarizes validly published prokaryotic taxonomic discoveries related to domesticated companion, agricultural, and farmed avian hosts for the calendar year 2024.

## MATERIALS AND METHODS

Per the International Committee on Systematics of Prokaryotes, validly published novel and revised taxa pertinent to prokaryotic species must satisfy two requirements set forth by the International Code of Nomenclature of Prokaryotes (17). First, original investigations are published in the *International Journal of Systematic and Evolutionary Microbiology* (IJSEM). An example relative to the aerobic Gram-negative bacillus *Bombella pluederhausensis* is provided in (18). In addition, type strains are to be deposited in recognized culture collections in two separate nations. As of January 2018, taxon descriptions in IJSEM require genome sequencing data relative to the type strain to be deposited in GenBank (19), with genome accession number included as part of the effective description. The similarity of this sequence should be assessed against related taxa.

As an alternative to primary publication in IJSEM, studies may be published in another journal, with later inclusion in IJSEM. One past example relative to bacteria derived from domestic veterinary material is the effective description of the aerobic Gram-positive bacillus *Paenibacillus mellifer* (20), with subsequent inclusion on an IJSEM Validation List (21). To be considered for inclusion on a Validation List, which is published six times per year, authors must submit a copy of the previously published manuscript to the editorial office of IJSEM for confirmation that all elements necessary for valid publication (including culture collection) have been met. It has been estimated that half of effectively published taxa outside IJSEM are not submitted to IJSEM for inclusion on Validation Lists (22).

Non-IJSEM journals that have recently published studies providing valid descriptions of domestic animal-derived novel taxa, which may be relevant to the practice of veterinary microbiology, include *Anaerobe*, *Antonie Van Leeuwenhoek*, *Archives of Microbiology*, *Cell Host & Microbe*, *Research in Microbiology*, *Scientific Reports*, *Systematic and Applied Microbiology*, and *Vector Borne Zoonotic Diseases*. Journals that have recently published studies reflecting revisions in prokaryotic taxonomy relative to domestic veterinary clinical material include *Antonie Van Leeuwenhoek* and *Systematic and Applied Microbiology*.

In the event of taxonomic reclassification, original taxon names validly published in IJSEM as primary papers or on Validation Lists may later be considered synonyms (e.g., by considering them as a heterotypic synonym of a name of the same rank, which has priority, or by proposing a new combination when transferring a species to another genus or when transferring a subspecies to another species). Moreover, Trujillo et al. (23) stated that once a taxon is validly published or included on a Validation List, it remains available for use as long as it is the correct name for the appropriate circumscription, position, and rank. Designations that are transferred to another taxon frequently do not relinquish a validly published status; the majority of prokaryotic nomenclature revisions are due to changes in taxonomic opinion. For example, the recently described *Borreliella burgdorferi* taxon designation (24), included on Validation List no. 163 (25), currently stands as a valid synonym to its correct designation of *Borrelia burgdorferi* (26). Table headers throughout this document include phrases such as “previous designation” and “revised designation” to reflect this paradigm.

All issues of IJSEM published from January 2024 through December 2024 (including six Validation Lists) were manually searched for original articles describing novel, validly published species taxonomy or accepted changes in taxonomic nomenclature. With the goal of consistency, only nomenclature with taxonomic status of “correct name” (per data curated by LPSN—List of Prokaryotic names with Standing in Nomenclature; https://lpsn.dsmz.de) was included. On rare occasion, taxa within Validation Lists published in 2024 (e.g., *Gemelliphila palaticanis* comb. nov. [27, 28]) were subsequently classified by LPSN as synonym designations (e.g., correct name *Gemella palaticanis* [29]) and thus were excluded. The audit was further filtered by organisms recovered from domestic animals. Included in the definition of domestic animals are animals found in agricultural settings, companion animals, and farmed avian species. In addition, novel species were isolated from honeybees or from honey/honeycomb. Honeybees are classified as livestock or food-producing animals by US federal government bodies, including the Food and Drug Administration (30). Associated organisms are included in this taxonomy summary due to the role of veterinary microbiology laboratories in providing culture and antimicrobial susceptibility testing results (per Clinical and Laboratory Standards Institute document VET06) for veterinarians, who may prescribe antimicrobials for veterinary feed directives in the treatment of honeybees.

## RESULTS AND DISCUSSION

A compilation of novel taxa recovered from domestic animal sources stratified by Gram morphology and oxygen growth requirement (when fully provided) is presented in Table 1. The phenotypic means of taxa organization within Table 1 is tailored to the practicing veterinary microbiologist. Taxonomic designations are presented in alphabetic order, rather than chronologic order, within each stratification. Table 2 provides taxonomic revisions for organisms originally recovered from domestic and companion animal sources.

**TABLE 1:** Novel, validly published bacterial species recovered from domestic (companion animal, agricultural, farmed avian) veterinary material reported from January 2024 to December 2024

Scientific name	Family	Source^*a*^	Growth Characteristics	Reference(s)
Gram-positive cocci				
*Aerococcus kribbianus* sp. nov.	*Aerococcaceae*	Isolates (2) from feces of pig in the Republic of Korea	Facultative, non-motile Gram-positive coccus; colonies cultivated on reinforced clostridial medium with 5% sheep blood; optimal growth at 37°C; positive for alkaline phosphatase, leucine arylamidase, acid phosphatase, maltose, β-glucuronidase; negative for galactosidase, glycerol, mannitol, mannose; strain-dependent reactions for sucrose, salicin	(31)
*Planococcus wigleyi* sp. nov.	*Caryophanaceae*	Feces from chicken in England	Limited phenotypic characterization provided in (32); Gram reaction and atmospheric requirement predicted by genus assignment	(33)
*Streptococcus caecimuris* sp. nov.	*Streptococcaceae*	Cecal content of specific pathogen- free mouse	Limited phenotypic characterization provided; Gram reaction and atmospheric requirement predicted by genus assignment	(34)^*b*^
Gram-positive bacilli				
*Arthrobacter gallicola* sp. nov.	*Micrococcaceae*	Feces from chicken in England	Limited phenotypic characterization provided in (32); Gram reaction and atmospheric requirement predicted by genus assignment	(33)
*Arthrobacter pullicola* sp. nov.	*Micrococcaceae*	Feces from chicken in England	Limited phenotypic characterization provided in (32); Gram reaction and atmospheric requirement predicted by genus assignment	(33)
*Bacillus norwichensis* sp. nov.	*Bacillaceae*	Feces from chicken in England	Limited phenotypic characterization provided in (32); Gram reaction and atmospheric requirement predicted by genus assignment	(33)
*Brevibacterium gallinarum* sp. nov.	*Brevibacteriaceae*	Feces from chicken in England	Limited phenotypic characterization provided in (32); Gram reaction and atmospheric requirement predicted by genus assignment	(33)
*Cellulomonas avistercoris* sp. nov.	*Cellulomonadaceae*	Feces from chicken in England	Limited phenotypic characterization provided in (32); Gram reaction and atmospheric requirement predicted by genus assignment	(33)
*Corynebacterium gallinarum* sp. nov.	*Corynebacteriaceae*	Feces from chicken in England	Limited phenotypic characterization provided in (32); Gram reaction and atmospheric requirement predicted by genus assignment	(33)
*Cytobacillus stercorigallinarum* sp. nov.	*Cytobacillaceae*	Feces from chicken in England	Limited phenotypic characterization provided in (32); Gram reaction and atmospheric requirement predicted by genus assignment	(33)
*Lactobacillus amylovorus* subsp. *animalium* subsp. nov.	*Lactobacillaceae*	Isolates (5) from weaning piglets, porcine feces, and bovine feces in Japan	Originally published as *Lactobacillus amylovorus* subsp. *animalis* (35); facultative, non-motile, catalase-negative, oxidase-negative, non-spore-forming Gram-positive bacillus; 1 to 2 mm diameter milky-white, round, glossy colonies on de Man-Rogosa-Sharpe (MRS) agar; optimal growth at 37°C; positive for D-lactose, cystine arylamidase; negative for D-mannitol, salicin, glycogen, gentiobiose, D-melibiose	(36)
*Ligilactobacillus hohenheimensis* sp. nov.	*Lactobacillaceae*	Jejunum of broilers Ross 308 in Germany	Facultative, non-spore-forming Gram-positive bacillus; propagated on MRS agar; positive for maltose, D-serine, D-fructose-6-phosphate, L-histidine, glucuronamide, sodium lactate, sodium butyrate; growth in Tween 40 and 8% NaCl; reported resistance to minocycline	(37)
*Limosilactobacillus avium* sp. nov.	*Lactobacillaceae*	Ileum of Brown Lohmann laying hens in Germany	Facultative, non-spore-forming Gram-positive bacillus; propagated on MRS agar; positive for trehalose, gentiobiose, glucose, raffinose, fructose, lactic acid, serine, sorbitol, glucuronamide, sodium butyrate, sodium lactate; growth in 8% NaCl; reported resistance to vancomycin, minocycline, nalidixic acid	(37)
*Limosilactobacillus difficilis* sp. nov.	*Lactobacillaceae*	Ileum of broilers Ross 308 in Germany	Facultative, non-spore-forming Gram-positive bacillus; propagated on MRS agar; positive for D-fructose, D-fucose, D-galactose, D-glucose, maltose, melibiose, raffinose, trehalose, L-rhamnose, D-serine, dextrin, turanose, inosine; growth in Tween 40; reported resistance to vancomycin, aztreonam, minocycline, nalidixic acid	(37)
*Limosilactobacillus galli* sp. nov.	*Lactobacillaceae*	Crop of Brown Lohmann laying hens in Germany	Facultative, non-spore-forming Gram-positive bacillus; propagated on gut microbiota medium; positive for maltose, sucrose, raffinose, lactic acid, serine, fructose, acetic acid, butyric acid, acetoacetic acid; growth in 8% NaCl, Tween 40; reported resistance to minocycline	(37)
*Limosilactobacillus pulli* sp. nov.	*Lactobacillaceae*	Ileum of Brown Lohmann laying hens in Germany	Facultative, non-spore-forming Gram-positive bacillus; propagated on poultry-feed agar; positive for dextrin, D-glucose, D-fructose, D-galactose, D-glucuronic acid, maltose, turanose, pectin, fucose, gentiobiose, glucuronamide, inosine, sucrose, stachyose, sodium lactate, sodium butyrate, D-serine, L-arginine; growth in 4% NaCl, Tween 40; reported resistance to vancomycin, minocycline, nalidixic acid	(37)
*Limosilactobacillus viscerum* sp. nov.	*Lactobacillaceae*	Ileum of Brown Lohmann laying hens in Germany	Facultative, non-spore-forming Gram-positive bacillus; propagated on MRS agar; positive for maltose, raffinose, lactose, D-glucose, D-mannitol, D-serine, acetoacetic acid, sodium butyrate, α-keto-glutaric acid; growth in Tween 40; reported resistance to minocycline	(37)
*Microbacterium commune* sp. nov.	*Microbacteriaceae*	Feces from chicken in England	Limited phenotypic characterization provided in (32); Gram reaction and atmospheric requirement predicted by genus assignment	(33)
*Microbacterium gallinarum* sp. nov.	*Microbacteriaceae*	Feces from chicken in England	Limited phenotypic characterization provided in (32); Gram reaction and atmospheric requirement predicted by genus assignment	(33)
*Microbacterium helvum* sp. nov.	*Microbacteriaceae*	Feces from cow in China	Aerobic, non-motile, non-spore-forming Gram-positive bacillus; smooth, circular, light yellow colonies on International *Streptomyces* Project 2 agar; optimal growth at 28°C; positive for urease, inositol, sucrose, raffinose, L-proline, adipic acid, potassium gluconate, C4 esterase, C8 esterase lipase, α-galactosidase, β-galactosidase, β-glucosidase, valine arylamidase; negative for Tweens 20, 40, 80 hydrolysis, H_2_S, glycine, L-arginine, alkaline phosphatase, amygdalin	(38)^*b*^
*Microbacterium pullorum* sp. nov.	*Microbacteriaceae*	Feces from chicken in England	Limited phenotypic characterization provided in (32); Gram reaction and atmospheric requirement predicted by genus assignment	(33)
*Oerskovia douganii* sp. nov.	*Promicromonosporaceae*	Feces from chicken in England	Limited phenotypic characterization provided in (32); Gram reaction and atmospheric requirement predicted by genus assignment	(33)
*Oerskovia gallyi* sp. nov.	*Promicromonosporaceae*	Feces from chicken in England	Limited phenotypic characterization provided in (32); Gram reaction and atmospheric requirement predicted by genus assignment	(33)
*Oerskovia merdavium* sp. nov.	*Promicromonosporaceae*	Feces from chicken in England	Limited phenotypic characterization provided in (32); Gram reaction and atmospheric requirement predicted by genus assignment	(33)
*Oerskovia rustica* sp. nov.	*Promicromonosporaceae*	Feces from chicken in England	Limited phenotypic characterization provided in (32); Gram reaction and atmospheric requirement predicted by genus assignment	(33)
*Paenibacillus gallinarum* sp. nov.	*Paenibacillaceae*	Feces from chicken in England	Limited phenotypic characterization provided in (32); Gram reaction and atmospheric requirement predicted by genus assignment	(33)
*Solibacillus faecavium* sp. nov.	*Caryophanaceae*	Feces from chicken in England	Limited phenotypic characterization provided in (32); Gram reaction and atmospheric requirement predicted by genus assignment	(33)
*Sporosarcina gallistercoris* sp. nov.	*Caryophanaceae*	Feces from chicken in England	Limited phenotypic characterization provided in (32); Gram reaction and atmospheric requirement predicted by genus assignment	(33)
*Sporosarcina quadrami* sp. nov.	*Caryophanaceae*	Feces from chicken in England	Limited phenotypic characterization provided in (32); Gram reaction and atmospheric requirement predicted by genus assignment	(33)
Gram-negative cocci				
*Moraxella oculi* sp. nov.	*Moraxellaceae*	Conjunctival swab from cow (Angus cross) with infectious keratoconjunctivitis in the United States	oxidase-positive, catalase-positive Gram-negative diplococcus (some observed as Gram-negative bacilli); 1 to 3 mm diameter γ-hemolytic, shiny gray, round, entire colonies on sheep blood agar when incubated in 35°C, 5% CO_2_ conditions; no growth on MacConkey agar; negative for indole, urease, arginine dihydrolase, lysine decarboxylase, ornithine decarboxylase, arabinose, mannose, mannitol, maltose, malate, *N*-acetyl-glucosamine, glucose fermentation	(39)
*Neisseria leonii* sp. nov.	*Neisseriaceae*	Archival isolates (3) derived from nose, lung, and liver of rabbits in France and Switzerland between 1972 and 2000	Aerobic, non-motile, oxidase-positive, catalase-positive, elongated Gram-negative diplococcus; small, round, shiny, translucent colonies on tryptic soy agar; optimal growth at 37°C, growth with or without 5% CO_2_; colonies non-hemolytic on blood-containing agar; positive for indole; negative for glucose, fructose, sucrose	(40)
Gram-negative coccobacilli and bacilli				
*Acinetobacter pecorum* sp. nov.	*Moraxellaceae*	Feces from chicken in England	Limited phenotypic characterization provided in (32); Gram reaction and atmospheric requirement predicted by genus assignment	(33)
*Apirhabdus apintestini* gen. nov., sp. nov.	*Enterobacteriaceae*	Gut of honeybee (*Apis mellifera*) in the United States	Facultative, motile, catalase-positive, oxidase-negative, Gram-negative bacillus; 1 to 2 mm diameter non-hemolytic, circular, opaque, glistening, convex, gray colonies on Columbia blood agar; optimal growth at 37°C; positive for D-mannitol; negative for urease, nitrate reduction, inositol, D-glucose, L-rhamnose; susceptibility to ampicillin, ceftriaxone, ciprofloxacin, gentamicin, tetracycline, trimethoprim-sulfamethoxazole; resistant to lincomycin	(41)
*Brevundimonas guildfordensis* sp. nov.	*Caulobacteraceae*	Feces from chicken in England	Limited phenotypic characterization provided in (32); Gram reaction and atmospheric requirement predicted by genus assignment	(33)
*Comamonas avium* sp. nov.	*Comamonadaceae*	Feces from chicken in England	Limited phenotypic characterization provided in (32); Gram reaction and atmospheric requirement predicted by genus assignment	(33)
*Escherichia whittamii* sp. nov.	*Enterobacteriaceae*	Feces from chicken in England	Limited phenotypic characterization provided in (32); Gram reaction and atmospheric requirement predicted by genus assignment	(33)
*Luteimonas colneyensis* sp. nov.	*Lysobacteraceae*	Feces from chicken in England	Limited phenotypic characterization provided in (32); Gram reaction and atmospheric requirement predicted by genus assignment	(33)
*Mannheimia indoligenes* sp. nov.	*Pasteurellaceae*	25 bovine isolates (including nine from rumen, six from tongue, one from uterus, three from pneumonia, three from placenta or products of pregnancy) characterized from Scotland, Germany, Belgium, Denmark	Non-motile, short, pleomorphic Gram-negative bacillus; majority of strains catalase-positive and oxidase-positive; 1.5 to 2.0 mm diameter circular, slightly raised, opaque, grayish, regular colonies on bovine blood agar incubated at 37°C; variable hemolysis, with 20% exhibiting β-hemolysis; neither hemin nor nicotinamide adenine dinucleotide required for growth; positive for indole (delayed reactivity), D-xylose, L-fucose, D-galactose, ONPG; negative for melibiose, β-glucosidase, α-fucosidase, α-galactosidase	(42)
*Moraxella haemolytica* sp. nov.	*Moraxellaceae*	Lung from dead goat with respiratory disease in China	Aerobic, oxidase-positive, catalase-positive Gram-negative bacillus; 1.0 to 1.5 mm diameter gray-white, convex, round β-hemolytic colonies on Columbia agar; optimal growth at 37°C; growth on MacConkey agar; positive for glucose, arabinose, potassium gluconate, malate; negative for mannose, maltose, mannitol, gelatin, *N*-acetyl-glucosamine; susceptible to ampicillin, amoxicillin-clavulanate, cefuroxime, cefotaxime, gentamicin, tetracycline, chloramphenicol	(43)^*c*^
*Paenalcaligenes faecalis* sp. nov.	*Alcaligenaceae*	Feces from chicken in China	Aerobic, motile, oxidase-positive, catalase-positive Gram-negative bacillus; beige, shiny, smooth, regular colonies on Luria-Bertani agar; isolate originally recovered on MacConkey agar; optimal growth at 28°C; growth in 10% NaCl; positive for urease; negative for gelatinase, amylase, indole, H_2_S, casease	(44)
*Pseudomonas kulmbachensis* sp. nov.	*Pseudomonadaceae*	Isolated from beef in slaughterhouse in Germany; second isolate derived from chicken breast	Aerobic, motile, oxidase-positive, catalase-positive, non-spore-forming Gram-negative bacillus; 3.2 mm diameter cream white, glossy, round, convex, non-hemolytic colonies on Columbia blood agar; optimal growth at 20–30°C; growth on Reasoner’s 2A agar; non-fluorescent on King B and cetrimide agars; positive for L-arabinose, L-arginine, D-galactose, D-fucose; negative for nitrate reduction, gelatin hydrolysis, alkaline phosphatase, urease, sucrose, trehalose, acetic acid, fructose, m-inositol, uridine, xylitol	(45)
*Pseudomonas paraveronii* sp. nov.	*Pseudomonadaceae*	Isolates (2) from beef in slaughterhouse in Germany	Aerobic, motile, oxidase-positive, catalase-positive, non-spore-forming Gram-negative bacillus; 1.3 mm diameter cream white, glossy, round, convex, non-hemolytic colonies on Columbia blood agar; optimal growth at 20-30°C; growth on Reasoner’s 2A agar; non-fluorescent on King B and cetrimide agars; positive for nitrate reduction, L-arabinose, arginine, D-arabitol, trehalose, alkaline phosphatase, C4 esterase, C8 esterase lipase, acid phosphatase; negative for gelatin hydrolysis, maltose, inositol, L-rhamnose, D-sorbitol, sucrose, xylitol, valine arylamidase, trypsin	(45)
*Psychrobacter communis* sp. nov.	*Moraxellaceae*	Feces from chicken in England	Limited phenotypic characterization provided in (32); Gram reaction and atmospheric requirement predicted by genus assignment	(33)
*Psychrobacter raelei* sp. nov.	*Moraxellaceae*	Peritoneal exudate from dog with peritonitis in Italy	Aerobic, non-motile, oxidase-positive, catalase-positive Gram-negative coccobacillus; 2 to 4 mm diameter moist, smooth, circular, slightly translucent, grayish, non-hemolytic colonies on tryptic soy agar with 5% sheep blood; optimal growth at 37°C; growth in 5% NaCl; colorless growth on MacConkey; positive for malate, indole, urease, nitrate reduction, sodium acetate; negative for 5-keto-D-gluconic acid, alkaline phosphatase, cystine arylamidase, adonitol, arabinose, D-glucose, inositol, mannitol, rhamnose, sorbitol, salicin, sucrose, gelatin	(46)
*Stenotrophomonas forensis* sp. nov.	*Lysobacteraceae*	Tracheal wash, transtracheal aspirate	Novel taxonomic designation within *Stenotrophomonas maltophilia* complex reflects isolates derived from *Equus caballus* previously classified as *S. maltophilia*	(47)
*Stenotrophomonas pennii* sp. nov.	*Lysobacteraceae*	Feces from chicken in England	Limited phenotypic characterization provided in (32); Gram reaction and atmospheric requirement predicted by genus assignment	(33)
*Ureibacillus galli* sp. nov.	*Caryophanaceae*	Feces from chicken in England	limited phenotypic characterization provided in (32); Gram reaction and atmospheric requirement predicted by genus assignment	(33)
Gram-positive anaerobes				
*Acetatifactor aquisgranensis* sp. nov.	*Lachnospiraceae*	Cecal content of specific pathogen-free mouse	Limited phenotypic characterization provided; Gram reaction and atmospheric requirement predicted by genus assignment	(34)^*d*^
*Acutalibacter caecimuris* sp. nov.	*Oscillospiraceae*	Cecum from C57BL/6 mouse in China	Anaerobic, motile, catalase-negative, non-spore-forming Gram-positive bacillus; smooth, irregular, cloudy-white colonies with rounded edges on modified Gifu anaerobic medium; optimal growth at 30°C; positive for *N*-acetyl-D-mannosamine, amygdalin, arbutin, D-fructose, L-fucose, gentiobiose, melibiose, L-serine, L-threonine; negative for maltose, D-xylose, L-arabinose, D-sorbitol, L-alanyl-L-threonine, urease	(48)
*Acutalibacter intestini* sp. nov.	*Oscillospiraceae*	Feces from C57BL/6 mouse in China	Anaerobic, motile, catalase-negative, non-spore-forming Gram-positive bacillus; smooth, uniform, glossy-white colonies with round edges on modified Gifu anaerobic medium; optimal growth at 35°C; positive for maltose, D-xylose, L-arabinose, D-sorbitol, L-alanyl-L-threonine; negative for *N*-acetyl-D-mannosamine, amygdalin, arbutin, D-fructose, L-fucose, gentiobiose, melibiose, L-serine, L-threonine, urease	(48)
*Adlercreutzia faecimuris* sp. nov.	*Eggerthellaceae*	Feces from BALB/c mouse	Anaerobic, non-motile, catalase-negative, oxidase-negative, non-spore-forming Gram-positive bacillus; pinpoint, circular, translucent, entire colonies on tryptic soy agar with 5% horse blood; optimal growth at 37°C; positive for arginine dihydrolase, arginine arylamidase, D-mannose, D-raffinose, naphthol-AS-BI-phosphohydrolase; negative for leucine arylamidase, histidine arylamidase, C4 esterase	(49)^*e*^
*Bifidobacterium apicola* sp. nov.	*Bifidobacteriaceae*	Isolates (2) from gut of honeybee (*Apis mellifera*) in China	Anaerobic, non-motile, catalase-negative, non-spore-forming Gram-negative bacillus; 0.2 mm diameter beige, smooth, convex, circular, entire colonies on modified MRS agar; optimal growth at 30–37°C; growth in 3% NaCl; positive for D-arabinose, L-arabinose, D-xylose, L-xylose, D-galactose, D-fructose, D-mannose, methyl-α-D-glucopyranoside, melibiose, acid phosphatase, cystine arylamidase; negative for maltose, sucrose, melezitose, L-fucose, pyruvate, C8 esterase lipase, *N*-acetyl-β-glucosaminidase	(50)
*Bifidobacterium apis* sp. nov.	*Bifidobacteriaceae*	Gut of honeybee (*Apis mellifera*) in China	Anaerobic, non-motile, catalase-negative, non-spore-forming Gram-positive bacillus; 1.5 mm diameter beige, convex, smooth, circular, entire colonies on modified MRS agar; optimal growth at 30–37°C; growth in 2% NaCl; positive for L-arabinose, D-galactose, cellobiose, melibiose, raffinose, Voges-Proskauer, pyruvate, hippurate, acid phosphatase, α-galactosidase; negative for D-xylose, D-fructose, D-mannose, amygdalin, maltose, sucrose, gentiobiose, turanose, C8 esterase lipase; reported resistance to neomycin (300 µg/mL), gentamicin (300 µg/mL), bacitracin (5 µg/mL),	(51)
*Clostridium butanoliproducens* sp. nov.	*Clostridiaceae*	Crop of Brown Lohmann laying hens in Germany	Anaerobic, spore-forming Gram-positive bacillus; propagated on tryptic soy agar; positive for D-glucuronic acid, D-glucose, D-fructose, D-glucuronic acid, L-rhamnose, D-serine, dextrin, pectin, aminobutyric acid; growth in 8% NaCl, Tween 40; reported resistance to vancomycin, aztreonam, minocycline, nalidixic acid	(37)
*Clostridium gallinarum* sp. nov.	*Clostridiaceae*	Feces from chicken in England	Limited phenotypic characterization provided in (32); Gram reaction and atmospheric requirement predicted by genus assignment	(33)
*Collinsella ureilytica* sp. nov.	*Coriobacteriaceae*	Feces from swine in the Republic of Korea	Originally published as *Collinsella urealyticum*; anaerobic, non-motile, catalase-negative, oxidase-negative, non-spore forming Gram-positive bacillus; convex, circular, white smooth colonies on tryptic soy agar with 5% sheep blood; optimal growth at 37°C; positive for urease, D-lactose, D-mannose, acid phosphatase, alkaline phosphatase, *N*-acetyl-β-glucosaminidase, serine arylamidase, negative for D-cellobiose, D-maltose, D-saccharose, salicin, D-trehalose, arginine dihydrolase, α-fucosidase, proline arylamidase	(52)^*c*^
*Faecalispora anaeroviscerum* sp. nov.	*Oscillospiraceae*	Ileum of Brown Lohmann laying hens in Germany	Anaerobic, spore-forming Gram-positive bacillus; propagated on postgate standard medium; positive for D-galactose, D-glucose, D-fructose, D-mannose, turanose, D-glucuronic acid, cellobiose, fucose, gentobiose, maltose, raffinose, rhamnose; reported resistance to vancomycin, aztreonam, minocycline, nalidixic acid	(37)
*Kribbibacterium absianum* gen. nov., sp. nov.	*Kribbibacteriaceae*	Isolates (2) from pig feces in the Republic of Korea	Anaerobic, non-motile, non-spore-forming Gram-positive bacillus; isolate originally recovered on BL agar, subsequent studies used organisms propagated on reinforced clostridial medium with 5% sheep blood; optimal growth at 37°C; growth in 2% NaCl; positive for urease, arginine dihydrolase, α-glucosidase, alkaline phosphatase, proline arylamidase, esculin, glutamyl-glutamic acid arylamidase; negative for α-galactosidase, β-glucosidase, β-galactosidase, *N*-acetyl-β-glucosaminidase	(53)
*Lactobacillus juensis* sp. nov.	*Lactobacillaceae*	Isolates (2) from honeybee gut (*Apis mellifera*) in China	Anaerobic, non-motile, catalase-negative, non-spore-forming Gram-positive bacillus; 1.1 to 1.3 mm diameter circular, milk white, smooth, convex, entire colonies on modified MRS medium; optimal growth at 37°C (growth is weak under aerobic conditions); growth in 3% NaCl; negative for L-arabinose, methyl-α-D-mannopyranoside, α-glucosidase; reported resistance to kanamycin, neomycin (50 µg/mL), streptomycin (100 µg/mL), tetracycline, gentamicin (50 µg/mL),	(54)
*Lactobacillus rizhaonensis* sp. nov.	*Lactobacillaceae*	Isolates (2) from honeybee gut (*Apis mellifera*) in China	Anaerobic, non-motile, catalase-negative, non-spore-forming Gram-positive bacillus; 1.1 to 1.2 mm diameter circular, milk white, smooth, convex, entire colonies on modified MRS medium; optimal growth at 37°C (growth is weak under aerobic conditions); growth in 3% NaCl; positive for L-arabinose, methyl-α-D-mannopyranoside, α-glucosidase; reported resistance to chloramphenicol, neomycin (5 µg/mL), streptomycin (5 µg/mL), tetracycline	(54)
*Olsenella absiana* sp. nov.	*Atopobiaceae*	Feces from pig in the Republic of Korea	Anaerobic, non-motile, non-spore-forming Gram-positive bacillus; isolate originally recovered on BL agar, subsequent studies used organisms propagated on reinforced clostridial medium with 5% sheep blood; optimal growth at 37°C; growth in 2% NaCl; positive for alkaline phosphatase, esterase, esterase lipase, trypsin, α-galactosidase, β-glucosidase, sucrose, salicin, xylose, arabinose, gelatin; negative for leucine arylamidase, β-glucuronidase, α-glucosidase, mannitol, lactose, cellobiose	(55)
*Peptostreptococcus equinus* sp. nov.	*Peptostreptococcaceae*	Feces from a female horse in the Republic of Korea	Anaerobic, non-motile, catalase-positive, oxidase-negative, non-spore forming, Gram-positive cocci; shiny, pale white, circular, convex colonies on brain heart infusion agar containing 5% sheep blood; optimal growth at 35°C; growth in 1% NaCl; positive for esculin hydrolysis, D-raffinose, glucose, D-mannose, salicin, mannitol, glycerol, trehalose, D-maltose, L-arabinose; negative for urease, indole, gelatin hydrolysis	(56)
*Turicibacter faecis* sp. nov.	*Turicibacteraceae*	Fecal sample from heart failure mouse model	Anaerobic, non-motile, catalase-negative, oxidase-negative, spore-forming Gram-positive bacillus; grayish-white, convex colonies on chocolate-blood agar; optimal growth at 37°C; positive for α-galactosidase, α-glucosidase, esculin hydrolysis, maltose, 5-keto-gluconate; negative for arginine arylamidase, glycine arylamidase, serine arylamidase, gelatin hydrolysis	(57)
Gram-negative anaerobes				
*Clostridium tanneri* sp. nov.	*Clostridiaceae*	Feces from domesticated alpaca (*Lama pacos*) in the United States	Anaerobic, non-motile, non-spore-forming Gram-negative bacillus; circular, opaque colonies on brain heart infusion agar with 5% sheep blood; optimal growth at 37°C; growth in 2% NaCl; positive for C4 esterase, C8 esterase lipase, acid phosphatase, naphthol-AS-BI-phosphohydrolase; negative for alkaline phosphatase, C14 lipase, leucine arylamidase, valine arylamidase, cysteine arylamidase, trypsin, α-galactosidase, α-glucosidase, β-glucosidase, *N*-acetyl-β-glucosaminidase	(58)
*Flintibacter muris* sp. nov.	*Oscillospiraceae*	Cecal content of specific pathogen-free mouse	Limited phenotypic characterization provided; Gram reaction and atmospheric requirement predicted by genus assignment	(34)^*b*^
*Fusibacillus kribbianus* gen. nov., sp. nov.	*Lachnospiraceae*	Feces from pig in the Republic of Korea	Anaerobic, non-motile, spore-forming, fusiform Gram-negative bacillus; cultivated on reinforced clostridial medium; optimal growth at 37°C; does not tolerate salinity; tolerates pH 10 conditions; positive for lactic acid, D-arabinose, L-arabinose, alkaline phosphatase, raffinose; negative for butyric acid, α-galactosidase, β-galactosidase, α-glucosidase, β-glucosidase, β-glucuronidase	(59)^*c*^
*Hallella absiana* sp. nov.	*Prevotellaceae*	Isolates (2) from pig feces in the Republic of Korea	Anaerobic, non-motile, non-spore-forming Gram-negative bacillus; cultivated on reinforced clostridial medium with optimal growth at 37°C; positive for α-galactosidase, β-galactosidase, α-glucosidase; variable reactivity for β-glucosidase, esterase; negative for β-glucuronidase, *N*-acetyl-β-glucosaminidase	(60)^*c*^
*Muribaculum caecicola* sp. nov.	*Muribaculaceae*	Cecal/colon content of an APC^min/+^ Msh2^-/-^ mouse	Limited phenotypic characterization provided; Gram reaction and atmospheric requirement predicted by genus assignment	(34)^*b*^
*Palleniella muris* sp. nov.	*Prevotellaceae*	Cecal/colon content of an APC^min/+^ Msh2^-/-^ mouse	Limited phenotypic characterization provided; Gram reaction and atmospheric requirement predicted by genus assignment	(34)^*b*^
*Prevotella communis* sp. nov.	*Prevotellaceae*	Isolates (2) from sheep rumen in Japan and Slovenia; also discerned from metagenomes from cattle and sheep in Scotland and New Zealand	Anaerobic, non-motile, short Gram-negative bacillus; colonial growth on M330 medium without rumen fluid supplementation; optimal growth at 34–42°C; does not tolerate bile; positive for gelatinase, esculin hydrolysis, α-galactosidase, α-arabinosidase; negative for α-glucosidase, β-glucosidase, α-fucosidase, tyrosine arylamidase	(61, 62)^*d*^
*Selenomonas ruminis* sp. nov.	*Selenomonadaceae*	Rumen fluid of Thai crossbred Saanen lactating goats	Anaerobic, motile, catalase-negative, non-spore-forming Gram-negative bacillus; 1 to 3 mm diameter opaque, white, circular, convex colonies on peptone yeast extract glucose agar; optimal growth at 37°C; positive for nitrate reduction, methyl red, starch hydrolysis, alkaline phosphatase, esterase, esterase lipase, acid phosphatase; acid formation from amidon, D-cellobiose, inositol, D-xylose; negative for L-rhamnose, D-ribose, D-trehalose	(63)^*f*^
Curved bacteria				
*Campylobacter californiensis* sp. nov.	*Campylobacteraceae*	Isolates (5) from cattle feces in the United States	Studied in association with *Campylobacter* spp. outbreak in raw/milk colostrum from a California dairy; microaerophilic, motile, oxidase-positive, catalase-negative, slightly-curved Gram-negative bacillus and coccoid cells; 1 to 2 mm diameter glistening, opaque, convex, circular, entire colonies on anaerobe basal agar with blood; growth at 37-42°C in microaerophilic conditions and at 30°C in anaerobic conditions; positive for nitrate, selenite, glycine; negative for alkaline phosphatase, hippurate, indoxyl acetate, urease; H_2_S on triple sugar iron agar is variable; resistant to nalidixic acid and cephalothin	(64)
*Campylobacter devanensis* sp. nov.	*Campylobacteraceae*	Isolates (37) from swine feces in Scotland, Slovenia, Switzerland	Microaerophilic, motile, oxidase-positive, catalase-positive Gram-negative spiral bacillus; 1 to 2 mm diameter opaque, glistening, circular, convex, entire colonies on anaerobe basal agar with blood after 3 days; growth at 37°C and 42°C (no growth at 30°C; no growth aerobically); positive for indoxyl acetate, nitrate reduction, selenite reduction, alkaline phosphatase; negative for urease, hippurate hydrolysis; resistant to nalidixic acid, cephalothin	(65)
*Campylobacter porcelli* sp. nov.	*Campylobacteraceae*	Isolates (6) from swine feces in Scotland	Microaerophilic, motile, oxidase-positive, catalase-positive Gram-negative curved bacillus; 1 to 2 mm diameter opaque, glistening, circular, convex, entire colonies on anaerobe basal agar with blood after 3 days; growth at 37°C and 42°C (no growth at 30°C; no growth aerobically); positive for nitrate reduction, selenite reduction, alkaline phosphatase; negative for urease, hippurate hydrolysis, indoxyl acetate; resistant to nalidixic acid, cephalothin	(65)
*Campylobacter vicugnae* sp. nov.	*Campylobacteraceae*	Isolates (9) from goat and alpaca (*Vicugna pacos*) feces in the United States	Microaerophilic, motile, oxidase-positive, catalase-positive Gram-negative spiral/curved bacillus; 1 to 2 mm diameter opaque, glistening, circular, convex, entire colonies on anaerobe basal agar with blood after 3 days; growth at 37°C and 42°C (no growth at 30°C; no growth aerobically); positive for nitrate reduction, alkaline phosphatase; negative for urease, hippurate hydrolysis, indoxyl acetate, selenite reduction; resistant to nalidixic acid; variable resistance to cephalothin	(65)
*Campylobacter sputorum* subsp. *bovis*, subsp. nov.	*Campylobacteraceae*	Isolates (6) from cattle feces in the United States	Microaerophilic, motile, oxidase-positive, catalase-positive Gram-negative curved/spiral bacillus, 1 to 2 mm diameter opaque, circular, convex colonies on anaerobe basal agar with blood; growth at 37 and 42°C, anaerobic at 37°C (no growth at 30°C; no growth aerobically); positive for alkaline phosphatase, production of H_2_S on TSI agar, selenite reduction, triphenyltetrazolium chloride reduction; negative for urease, indoxyl acetate, hippurate, nitrate reduction; resistant to cephalothin and nalidixic acid	(66)
Anaerobic bacteria not characterized by Gram reaction				
*Anaerocaecibacter muris* gen. nov., sp. nov.	*Pumilibacteraceae*	Filtered fecal suspension from specific pathogen-free mouse	Limited phenotypic characterization provided	(34)^*d*^
*Anaerotardibacter muris* gen. nov., sp. nov.	*Eggerthellaceae*	Gut content of specific pathogen-free mouse	Limited phenotypic characterization provided	(34)^*d*^
*Pumilibacter intestinalis* gen. nov., sp. nov.	*Pumilibacteraceae*	Cecal suspension of specific pathogen-free mouse	Limited phenotypic characterization provided	(34)^*d*^
*Pumilibacter muris* gen. nov., sp. nov.	*Pumilibacteraceae*	Cecal suspension of specific pathogen-free mouse	Limited phenotypic characterization provided	(34)^*d*^

^*a*^ Number of isolates from clinical source is 1, except where shown in parentheses.

^*b*^ Validly published by inclusion in Validation List no. 216 (67).

^*c*^ Validly published by inclusion in Validation List no. 217 (68).

^*d*^ Validly published by inclusion in Validation List no. 215 (28).

^*e*^ Validly published by inclusion in Validation List no. 220 (69).

^*f*^ Validly published by inclusion in Validation List no. 218 (70).

**TABLE 2:** Revised bacterial taxon relative to domestic (companion animal, agricultural, farmed avian) veterinary material from January 2024 to December 2024

Previous designation	Revised designation	Other information	Reference
Gram-positive cocci			
*Jeotgalicoccus pinnipedialis*	*Phocicoccus pinnipedialis* comb. nov.	Initial description of the *J. pinnipedialis* taxon from southern elephant seal (*Mirounga leonina*) found in (71); incidence in pigs documented in (72)	(27)^*a*^
*Kocuria polaris*	*Kocuria rosea* subsp. *polaris* comb. nov.	Initial description of the *K. polaris* taxon from an environmental source found in (73); incidence in calves documented in (74)	(75)
*Streptococcus suis*	*Streptococcus suis* subsp. *suis* subsp. nov.	Subspecies created following the valid publication of *Streptococcus suis* subsp. *hashimotonensis* subsp. nov (76). ; initial description of *S. suis* as a porcine pathogen found in (77)	(76)^*b*^
Gram-positive bacillus			
*Bacillus badius*	*Pseudobacillus badius* comb. nov.	Initial description of the *B. badius* taxon found in (78) and accepted (79); incidence in poultry documented in (80)	(81)^*b*^
Gram-negative coccobacillus			
*Pasteurella caecimuris*	*Rodentibacter caecimuris* comb. nov.	Initial description of the *P. caecimuris* taxon from laboratory mice found in (82); previous homotypic synonym designation of *Rodentibacter heylii* published in (83)	(84)
Obligate intracellular bacterium			
*Chlamydophila caviae*	*Chlamydia caviae* comb. nov	Initial valid publication of the *Chlamydophila caviae* taxon found in (85); pathogenic incidence in guinea pigs documented in (86)	(87)^*c*^

^*a*^ Validly published by inclusion in Validation List no. 215 (28).

^*b*^ Validly published by inclusion in Validation List no. 220 (69).

^*c*^ Validly published by inclusion in Validation List no. 218 (70).

A total of 28 taxa itemized in Table 1 came from two publications (32, 33) encompassing largely genotypic findings from chicken gut microbiome investigations. Gram morphology data were not available; thus, classification of these organisms within Table 1 is made on the basis of historic knowledge of Gram reaction and atmospheric requirement (one obligate anaerobic organism postulated out of the 28 taxa) of their assigned genus. A third publication (34) documented nine additional taxa derived from a laboratory murine host that were characterized in similar fashion. Four novel genus designations from Afrizal et al. resulted in the creation of a category within Table 1, demarcating anaerobic species with an unknown Gram stain reaction.

### Novel taxa

Numerous novel organisms were identified and characterized in 2024. Because of the significant number of organisms identified through microbiome research (Table 1), deeper discussion of novel organisms in this review will focus on those associated with disease in domestic and agricultural animals or derived from outbreaks in humans.

Novel organisms within the Gram-negative cocci grouping were *Moraxella oculi* sp. nov. (39), isolated from a cow with infectious bovine keratoconjunctivitis (IBK) but distinct from *Moraxella bovis* and *Moraxella bovoculi*, and *Neisseria leonii* sp. nov. (40), isolated from rabbits. IBK is a leading ocular disease in cattle, characterized by conjunctivitis, keratitis, corneal ulcer, potential rupture of the eye, and blindness (88). *M. bovis* is the primary cause of IBK, but *M. bovoculi* can also be isolated from corneal ulcers in cattle. Virulence factors associated with lesion production include cytotoxin RTX that causes corneal epithelial cell death and a type IV pilin *pilA* that is necessary for bacterial attachment (88). *M. oculi* was isolated from a cow with IBK, was non-hemolytic on blood agar (unlike *M. bovis* and *M. bovoculi*), lacked RTX, and did not assimilate glucose, arabinose, mannose, mannitol, *N*-acetyl-glucosamine, maltose, or malate (39). *N. leonii* was described from *Neisseriaceae* isolates collected from rabbits between 1972 and 2023 and deposited in the collection of the Institut Pasteur. One strain was isolated in 1972 from the liver of a kit, one in 1981 from the lung of a rabbit (both in France), and one in 2000 from the nose of a rabbit in Switzerland (40). No other information about the rabbits from which the isolates were collected was available, so it is unknown if the organism was associated with disease. Despite this, multiple potential virulence factors were identified in the genomes of the three isolates, including determinants associated with iron uptake, stress adaptation, and adhesion (40).

Within the Gram-negative coccobacilli and bacilli, there were three noteworthy organisms, namely *Mannheimia indoligenes* sp. nov.*, Moraxella haemolytica* sp. nov., and *Stenotrophomonas forensis* sp. nov. When genus *Mannheimia* was initially characterized (along with the renaming of *Pasteurella haemolytica* as *Mannheimia haemolytica*) in 1999, several biogroups within the new genus remained unnamed, including an indole-positive cluster similar to *Mannheimia varigena* within clade V of *Mannheimia* (89). Unlike *M. varigena*, *M. indoligenes* (42) is indole-positive, but the test requires incubation for one to two weeks. At 24-hour incubation, the indole test is negative for both *M. varigena* and *M. indoligenes*, thus making definitive identification using biochemical testing impractical for most clinical laboratories. Differentiation between *M. indoligenes* and other *Mannheimia* spp. was possible based on MALDI-TOF MS analysis or sequencing of the 16S rRNA gene (42). Both *M. varigena* and *M. indoligenes* can be differentiated from *M. haemolytica* based on negative ONPG, α-fucosidase, and 2-nitrophenyl α-L-fucopyranoside testing. For veterinary microbiology laboratories that may not have access to conventional biochemical testing, it should be noted that, on occasion, initial MALDI-TOF MS or 16S rRNA characterization of an isolate may need to be augmented by whole genomic sequencing.

In addition to the effective and valid publication of *M. indoligenes*, a new taxon within *Moraxellaceae* was recognized. Not to be confused with *Mannheimia haemolytica, Moraxella haemolytica* sp. nov. was isolated from the lung of a deceased goat with respiratory disease (43). The goat was part of a 176-animal herd in Southwest China that experienced morbidity in nine goats, with eventual mortality in four of the nine. Clinical signs in the goats included elevated temperature, cough, dyspnea, and purulent nasal discharge with gross lesions at necropsy that included pulmonary hemorrhage and purulent secretions within the trachea. *M. haemolytica* was isolated from the lung of a female, 14-month-old goat. Based on 16S rRNA sequencing, this Gram-negative bacillus was most closely related to *Moraxella caprae*. Phenotypically, *M. haemolytica* produced β-hemolysis and was able to assimilate glucose but was negative for utilization of mannose, maltose, and mannitol and unable to ferment glucose. While *Pasteurella* spp. and *Mannheimia* spp. are facultative anaerobes, *Moraxella* species are strictly aerobic (90).

*Stenotrophomonas maltophilia* is recognized as causing opportunistic infections in most mammalian species and is most notable as a multidrug-resistant organism (90). A novel *Stenotrophomonas* species was isolated as a contaminant in viral transport media at the District of Columbia Department of Forensic Sciences during the COVID-19 public health emergency (47). During characterization and phylogenetic analysis, genetically related isolates were identified in the National Center for Biotechnology Information (NCBI) GenBank. Among these isolates were those derived from tracheal washes in six horses in France (91). Related isolates came from all continents, except Africa and Antarctica (47). Biochemically, *S. forensis* sp. nov. can generate variable test results depending on the commercial test system used. The authors suggest the use of “*S. maltophila* complex” for reporting under these circumstances “to reduce confusion in clinical settings” (47).

There were several new additions to the *Campylobacter* genus, with most of these identified from animal feces; however, *Campylobacter californiensis* sp. nov. was studied as a result of infections from raw milk in humans (64). Other additions to *Campylobacter* spp. included *Campylobacter devanensis* sp. nov. and *Campylobacter porcelli* sp. nov. (both from swine feces), *Campylobacter vicugnae* sp. nov. from goat and alpaca feces (65), and *Campylobacter sputorum* subsp. *bovis*, subsp. nov. from bovine feces (66). *Campylobacter* spp. can be divided into those that grow at 42°C and those that do not. *C. californiensis* was isolated from feces from dairy cows collected during a raw milk/colostrum-associated *Campylobacter* outbreak in people in late 2007, as well as from cattle and swine feces collected during an epidemiologic survey of farms and ranches in 2009 and 2010 (92, 93). Nine isolates were initially identified as *Campylobacter* spp. based on 16S rRNA gene sequences, but their identity could not be resolved to the species level, although results suggested that they were related to *Campylobacter mucosalis* and *Campylobacter concisus* (64). Further characterization based on *atpA* determined that all nine isolates formed a novel clade most closely related to *C. mucosalis. C. californiensis* can be differentiated from other motile, catalase-negative *Campylobacter* spp. based on its inability to grow in a 30°C microaerophilic environment and its inability to grow under aerobic conditions at any temperature. *C. californiensis* is oxidase-positive but negative for alkaline phosphatase, hippuricase, urease, and does not hydrolyze indoxyl acetate. Additionally, *C. californiensis* is resistant to cephalothin and nalidixic acid (64).

*C. devanensis*, *C. porcelli,* and *C. vicugnae* are related species, similar to *Campylobacter lanienae*, within the *Campylobacter fetus* group (65). All three species are motile, catalase- and oxidase-positive, and positive for nitrate reduction. Similar to *C. lanienae*, the three novel species are deficient in selenium metabolism, but, unlike *C. lanienae*, the three taxa do not produce alpha hemolysis on anaerobe basal agar supplemented with 5% lysed horse blood. Hydrolysis of indoxyl acetate and reduction of selenite can further differentiate the three novel species.

A novel subspecies of *Campylobacter sputorum* was identified from cattle feces collected in California between March 2009 and October 2010 (66). *C. sputorum* is closely related to *C. mucosalis* and historically included three biovars (sputorum, faecalis, and paraureolyticus), which could be differentiated by catalase and urease testing (94). The newly described *Campylobacter sputorum* subsp. *bovis* subsp. nov. is catalase-positive, urease-negative, and alkaline phosphatase-positive, differing from the *C. sputorum* subsp. *sputorum* subsp. nov. designation described by Miller *et al*. (66), which is alkaline phosphatase-negative. Currently, the role of each of the five newly described *Campylobacter* spp. in veterinary disease is not well understood, but the recognition of their host range and geographic distribution may aid our understanding of these bacteria.

Of further interest are two Validation List entries from throughout 2024 (67, 69) that have since reverted to synonym status and are not included in Table 1. *Corynebacterium ramonii* sp. nov. (67, 95) is closely related to the *Corynebacterium ulcerans* taxon that can cause diphtheria-like infections in humans with animals as a potential reservoir (96–98). *C. ulcerans* can harbor a toxin determinant similar to that of *Corynebacterium diphtheriae* on either a corynephage or a pathogenicity island (99, 100). Infections in people present with respiratory clinical signs or cutaneous lesions similar to lesions observed in animals (101–107). Historically, two distinct lineages of *C. ulcerans* have been recognized: lineage 1 is most common, while lineage 2 is encountered infrequently (95).

A total of fourteen lineage 2 isolates were identified in the French National Reference Centre collection of 335 *C*. *ulcerans* isolates collected between 2018 and 2022. These isolates were compared with lineage 1 isolates using MALDI-TOF MS, whole genome sequencing, and phenotypic tests. A phylogenetic tree of the genomes for the *C. diphtheriae* complex was generated, demonstrating that lineage 2 isolates are distinct from lineage 1 (95). On MALDI-TOF MS, a peak specific to lineage 2 isolates at 5405.40 m/z was absent in lineage 1 and was also not present in the majority of other *C. diphtheriae* species complex members, except for occasional isolates of *Corynebacterium rouxii* (95). The presence of this peak did not alter the MALDI-TOF MS identification score. No phenotypic differences were noted by the authors (95). Both lineage 1 and lineage 2 possessed toxin-positive isolates. At the time, lineage 2 isolates were proposed to be a separate species, *C. ramonii*, named after the French veterinarian Gaston Ramon who invented the diphtheria toxoid vaccine (95). The lack of phenotypic differences and limited MALDI-TOF MS spectral differences could make delineation of *C. ramonii* from *C. ulcerans* difficult in the clinical laboratory, but recognition of the potential for both *C. ramonii* and *C. ulcerans* to harbor a toxin gene is significant (95). This is of concern due to the potential for disease in animals and people (101–107).

Historically, the taxonomic status of *Borrelia hermsii* was based on its association with the soft tick vector, *Ornithodoros hermsi*, similar to other relapsing fever spirochetes *Borrelia turicatae* and *Borrelia parkeri* being associated with *Ornithodoros turicata* and *Ornithodoros parkeri*, respectively. This long-term association of a single *Borrelia* species with each tick vector existed in part because of the historic inability to cultivate the organisms *in vitro*. With the development of chemically defined Kelly’s liquid media in 1971, isolation of the relapsing fever spirochetes became possible, and molecular characterization of these organisms has followed the development, expansion, and maturation of molecular techniques over the past 50 years (108, 109).

Recently, multilocus sequencing found that *B. hermsii* is comprised of two distinct groups, genomic group I and genomic group II, which were granted taxonomic status of *B. hermsii sensu stricto* and *Borrelia nietonii* sp. nov. (108), respectively, in a 2024 publication (69). As with *Borrelia burgdorferi*, relapsing fever spirochetes contain an oligopeptide transport system that includes the *oppA1* and *oppA2* determinants (108). *B. nietonii* harbors a third oligopeptide binding gene, *oppA3*, that encodes a predicted 547 amino acid protein. Also described is the presence of adenine deaminase (*adeC*) in *B. nietonii* that is present as a fragmented pseudogene in *B. hermsii sensu stricto* (108). Schwan et al. hypothesize that *B. nietonii* may be able to utilize more nutrients from the host, giving it a competitive advantage over *B. hermsii sensu stricto* due to the presence of the additional OppA binding protein and adenine deaminase, which provides an additional pathway for conversion of adenine to hypoxanthine. The geographic distribution for *B. nietonii* and *B. hermsii sensu stricto* is limited to western North America and should be considered in animals and people with relapsing fever (108–110). The presence of two unique genes should make it possible to develop molecular diagnostic tests to differentiate patients infected with *B. nietonii* and *B. hermsii sensu stricto*, which will allow further characterization of the organisms and disease.

### Taxonomic revisions

There were limited taxonomic revisions for organisms isolated from domestic animals during 2024 (Table 2). Within Gram-positive cocci, nomenclature revisions included *Jeotgalicoccus pinnipedialis* to *Phocicoccus pinnipedialis* comb. nov.*, Kocuria polaris* to *Kocuria rosea* subsp. *polaris* comb. nov., and *Streptococcus suis* to *Streptococcus suis* subsp. *suis* subsp. nov. Genetic analysis (27) revealed that species within the genus *Jeotgalicoccus* formed two distinct groups comprising a main clade with a single amino acid insertion in a glutamine synthetase family protein and a second group that included two species, *J. pinnipedialis* (originally isolated from the southern elephant seal *Mirounga leonina* [71] and later from pigs [72]) and *Jeotgalicoccus schoeneichii*. A two-amino acid deletion in the DNA polymerase III PolC-type protein differentiated these species from other members. The authors proposed that these two species form a new genus, *Phocicoccus*, named after the original source for *J. pinnipedialis*. Members of the new genus can be differentiated from other genera by the presence of indels in nine different genes. *P. pinnipedialis* incidence in pigs has been cited as a source of the antimicrobial resistance gene *cfr*, which confers florfenicol resistance (72).

*K. polaris* was originally described from the environment (73) and identified in the metagenome of the teat apex in primiparous, organically reared dairy cows from Colorado, Minnesota, New Mexico, and Texas (74). Analysis of the 16S rRNA gene and core protein sequences of members of *Kocuria* spp. and *Rothia* spp. indicated that *K. polaris* should be considered a subspecies of *K. rosea. K. rosea* subsp. *polaris* can be differentiated from *Kocuria rosea* subsp. *rosea* comb. nov. by its ability or preference to grow at lower temperatures but not at 37°C, its ability to produce acid from D-glucose, D-mannitol, L-rhamnose, and D-xylose, and its utilization of D-maltose, adonitol, D-melibiose, meso-inositol, lactose, pyruvate, inulin, L-arginine, L-aspartic acid, L-leucine, and L-phenylalanine as carbon sources, but not L-arabinose or L-asparagine (73). Although *K. rosea* subsp. *polaris* was not directly isolated from cattle, its finding in the metagenome of the teat is both of interest due to the importance of the dairy industry and plausible because of the cooler temperature at which the teat apex is likely to be maintained relative to body temperature, although the seasonality of the study is unknown (111).

Perhaps the most significant nomenclature change within Gram-positive cocci was the revision of *Streptococcus suis* to *S. suis* subsp. *suis* (76) in recognition of the initial description of *Streptococcus suis* subsp. *hashimotonensis* subsp. nov. (112), which was isolated from people in Japan with bite wounds from boars. *S. suis* subsp. *hashimotonensis* is Lancefield group A positive, similar in appearance to *S. suis*, and is identified as *S. suis* by some MALDI-TOF MS platforms with a score greater than 2.3. *S. suis* subsp. *suis* can be differentiated from *S. suis* subsp. *hashimotonensis* by its acidification of tagatose (76). This organism has the potential to be found in the oral cavity of swine or associated with bite wounds from swine in other animals, such as dogs.

In other Table 2 groupings, *Bacillus badius* (a probiotic organism) was revised to *Pseudobacillus badius* comb. nov., *Pasteurella caecimuris* was revised to *Rodentibacter caecimuris* comb. nov., and the correct designation of *Chlamydophila caviae* is now *Chlamydia caviae* comb. nov. Of these, *C. caviae* is the only one associated with disease, as both *P. badius* and *R. caecimuris* are currently only found within microbiota of their host species. In guinea pigs, *C. caviae* causes conjunctivitis, respiratory infections, anorexia, weight loss, and abortions (86, 113). It has also been associated with respiratory infections, including severe community-acquired pneumonia in humans (86, 114, 115).

## CONCLUSIONS

Taxonomic revisions develop a greater understanding of how bacteria are related, thereby bringing greater depth to biologic knowledge and offering potential insights into mechanisms of disease and opportunities for novel treatment. Although not discussed in depth here, the continued efforts to name organisms identified in gastrointestinal microbiome studies further underpin efforts at a greater understanding of the interconnectedness of animals and bacteria and the ecological niches they inhabit.
